# The Effectiveness and Feasibility of Palliative Care via Video Telemedicine for Patients with Advanced Cancer: A Nonrandomized Prospective Clinical Trial Comparing Combination of Telemedicine and in-Person Care with in-Person Care Alone

**DOI:** 10.1089/pmr.2024.0053

**Published:** 2024-11-08

**Authors:** Akihiko Chida, Yasuo Hamamoto, Kenro Hirata, Yasunori Sato, Eiichiro So, Shotaro Kishimoto, Satoko Noguchi, Sara Horie, Yuki Saito, Keitaro Shimozaki, Kai Tsugaru, Kazuhiro Togasaki, Kenta Kawasaki, Hideyuki Hayashi, Takanori Kanai

**Affiliations:** ^1^ Division of Gastroenterology and Hepatology, Department of Internal Medicine, Keio University School of Medicine, Tokyo, Japan.; ^2^ Keio Cancer Center, Keio University School of Medicine, Tokyo, Japan.; ^3^ Department of Preventive Medicine and Public Health, Keio University School of Medicine, Tokyo, Japan.; ^4^ Genomics Unit, Keio Cancer Center, Keio University School of Medicine, Tokyo, Japan.

**Keywords:** FACT-G, online medical care, palliative care, QOL, video telemedicine

## Abstract

**Background:**

Palliative care has been shown to be effective for patients with advanced cancer; however, the best approach to deliver palliative care needs to be considered. We hypothesized that a combination of palliative care via video telemedicine (TMD) and usual outpatient treatment would improve patients’ quality of life (QOL) and ameliorate depression and prognosis.

**Methods:**

Patients with advanced cancer who terminated treatment with chemotherapy were enrolled. Patients who could perform videoconferencing were assigned to the TMD group and those who could not perform were assigned to the (no TMD; control) group. The primary endpoint was QOL, which was evaluated using the difference in the Functional Assessment of Cancer Therapy-General (FACT-G) scores between baseline and one-month follow-up. Secondary endpoints included depression, measured using the Center for Epidemiological Studies Depression (CES-D) scale, overall survival, and patient satisfaction.

**Results:**

Fifty patients were included in this study (25 in each group). A comparison of measures of QOL at one month showed that the TMD group maintained a better QOL. FACT-G decreased by 0.21 and 5.96 points in the TMD and no TMD groups, respectively (*p* = 0.047). The TMD group maintained better QOL. There were trends suggesting improvements in depression and survival which did not reach significance. CES-D increased by 0.92 and 3.50 points in the TMD and no TMD groups, respectively (*p* = 0.26). Median survival time was 7.82 (3.30–14.59) and 6.37 (2.33–11.04) months in the TMD and no TMD groups, respectively. The results of the Patient Satisfaction Questionnaire suggest that video TMD could be a feasible tool in palliative care.

**Conclusion:**

Video TMD is effective in maintaining the QOL of patients with advanced cancer and is feasible based on patient satisfaction. It is desirable to further evaluate the usefulness of palliative care using video TMD and evaluate its clinical applications.

## Introduction

In recent years, there has been growing interest in the physical and psychosocial needs of patients with cancer. Particularly, accumulated evidence has shown that palliative care interventions improve quality of life (QOL) and prognosis, as well as ameliorate depression in patients with advanced cancer.^1–4^ Although palliative care must be provided to patients with advanced cancer, more detailed investigations need to be held on how to deliver palliative care to maximize its usefulness.

There are various methods of physical and psychological care intervention, and online medical care is one of them. With the continuous development of Information and Communication Technology (ICT), doctor-to-patient telemedicine (TMD), in which health care services are provided remotely to patients via audiovisual systems, has attracted attention. Initially, TMD was used in resource-poor settings, such as rural areas;^5^ however, as ICT equipment became more widespread, TMD gained prominence in all regions. TMD has been demonstrated to reduce the needs for emergency hospitalization^6^ and improve satisfaction among elderly and homebound patients. Many studies^6–8^ have been conducted in areas such as mental health, rehabilitation, palliative care, and findings from some of these studies have guided the implementation of TMD in clinical practice.

Before 2019, relatively few studies looked at the potential impact of TMD in the care of patients who receive palliative care. One study evaluated a web-based method for systematically collecting patient-reported symptoms during chemotherapy treatment and automatically alerting clinicians when symptoms are severe or worsening.^9^ This study found that the web-based intervention group had fewer patients visiting emergency rooms, improved health-related quality outcomes, decreased hospitalizations, and improved survival.^9^ However, TMD using videoconferencing had not been actively implemented.

The needs for social distancing and patient safety during the COVID-19 pandemic have played a significant role in the emergence of video TMD. The majority of the patients in supportive and palliative care clinics have advanced cancer or other chronic conditions and may be immunocompromised. It was critical to safeguard these patients from exposure to COVID-19, while continuing to help with their symptom management, access to opioids, and other supportive care needs.^10^ In such a situation, it was reported that repeated video-based online clinics have improved the experience of clinicians, technical problems have decreased, and device improvements have made for a smoother and more effective medical treatment.^11^ Various models for ideal delivery of community palliative care were discussed, such as incorporating both in-person visits and virtual care, or using two different types of care for each patient.^12–14^

However, most reports are from North America by nurses,^15^ few prospective studies have been conducted on physician-led online medical services with video TMD in palliative care of cancer patients. The needs for palliative care using TMD are expected to increase in Japan in the future due to the aging society and reduced resources. We considered that patients who no longer need to make outpatient visits for anticancer treatment and whose condition is not bad enough to stay in an inpatient facility would be one of the targets for successfully utilizing online medical care.

Indicators to measure the effectiveness of palliative care include QOL, depression, and prognosis.^1,3^ The Functional Assessment of Cancer Therapy-General (FACT-G), which was developed by Cella et al. as a basic QOL questionnaire for cancer patients,^16^ is an internationally used index for QOL research, and the reliability and validity of its Japanese version have been confirmed.^17^ It comprises four subscales: physical, social/family, psychological, and functional. The FACT-G is unique in that it has only one section of psychological assessment and can also assess physical or social aspects. Additionally, the Center for Epidemiological Studies Depression Scale (CES-D),^18^ an index of depression, was developed by the U.S. National Institute of Mental Health for epidemiological studies: a higher score on the 60-point scale indicates a depressive tendency. Additionally, as an indicator of palliative care feasibility, we evaluated patient satisfaction using the patient satisfaction questionnaire, which was modified from a previous study by Perri et al.^19^ Therefore, this study aimed to assess the effectiveness and feasibility of palliative care via video TMD for patients with advanced cancer.

We hypothesized that a combination of palliative care via video TMD with outpatient treatment would improve patients’ QOL and ameliorate depression, ultimately improving prognosis. We conducted a prospective study to evaluate the effect of palliative care via video TMD on these indicators using the research instruments explained above.

## Materials and Methods

### Study design

This nonrandomized clinical trial was conducted at Keio University Hospital. Participants were patients who visited the outpatient care between July 2020 and June 2022. The study inclusion criteria were the Eastern Cooperative Oncology Group Performance Status (ECOG PS) scores 0–3, ages ≥20 years, and advanced cancer patients who terminated chemotherapy due to disease progression or deterioration of general condition and planned to receive best supportive care (BSC). Patients who could not complete the questionnaire were excluded. Among the enrolled patients, those who had access to a device that supported video conferencing as well as broadband Internet were assigned to the TMD group, and those who didn’t have were assigned to the no TMD group as a control arm. Reasons for not being able to participate in videoconferencing included not having ICT devices such as smartphones and Internet access. The TMD group received the following interventions, and the no TMD Group also received the usual BSC at a hospital about once a month.

### Intervention

**FIG. 1.**
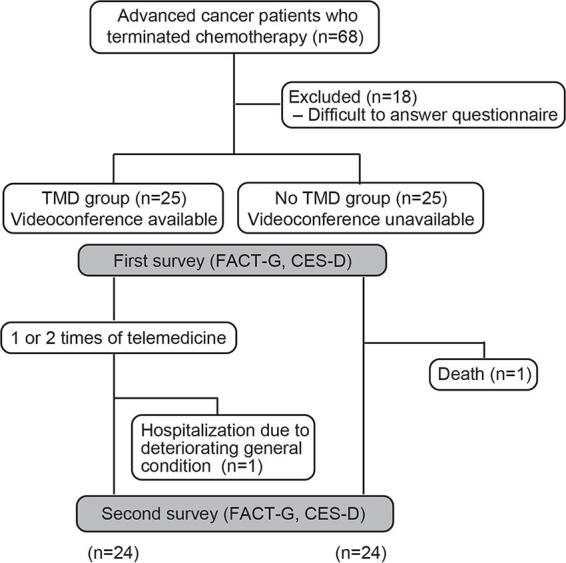
Flow diagram of patient selection and analysis. TMD, Telemedicine.

Patients in both groups underwent two face-to-face outpatient consultations within one month intervals for BSC by an oncologist. In the TMD group, the patients underwent one or two online medical examinations between their outpatient appointments per one month (Fig. 1). One oncologist and one nurse conducted the TMD. The following health services were provided during the videoconferencing:

1. Assessment and management of symptoms: We evaluated the symptoms of cancer, such as pain, nausea, vomiting, fatigue, and abdominal distention, and add prescriptions when necessary.
2. Mental distress and support: The patient’s mental distress, including insomnia and anxiety, must be heard, and ways to cope must be determined in partnership with the patient. If the patient has symptoms, prescriptions should be added as needed.
3. Troubles in daily life: In patients with terminal cancer, physical limitations arise as the disease progresses and nutritional status deteriorates. We review the situation in the home via videoconference and consider whether improvements to the home environment, such as the introduction of a care bed, could make life easier.
4. Family and caregiver concerns: In cases where family members participate in TMD, problems from the caregiver’s perspective are also discussed. We listen to the caregiver’s feelings and consider ways to reduce the caregiver’s physical and psychological burden.
5. Advance care planning (ACP): We discuss what patients value in their lives and where they would like to receive end-of-life care.

### Device for videoconferencing

In the TMD group, TMD was provided via the videoconference system, CLINICS, by one oncologist, and one nurse. The medical staff used a computer located in the medical facility, and the patients used a smartphone, tablet, or computer to attend the video conference. Some patients participated alone, while others were assisted by family members or other caregivers.

### Assessment items

As mentioned above patients in both groups underwent two face-to-face outpatient consultations within one-month intervals. The patients responded to the FACT-G: QOL, Center for Epidemiological Studies-Depression (CES-D: depression) at each outpatient visit. Patients in the TMD group responded to the patient satisfaction questionnaire described below (Fig. 1).

### Patient satisfaction questionnaire

The patient satisfaction questionnaire was used to measure satisfaction with online medical care. The questionnaire was modified from a previous study by Perri et al.,^19^ which examined satisfaction with online medical care during early palliation. In addition to technical sections, the questionnaire allowed for a comprehensive evaluation of the feasibility of online palliative care. Patient satisfaction was rated using the following eight questions with a 5-point Likert scale (5 strongly agree, 4 agree, 3 neutral, 2 disagree, and 1 strongly disagree). (1) I could see other videoconferencing participants clearly. (2) I could clearly hear other videoconferencing participants. (3) I felt that my conversations during the videoconferencing session were private. (4) I feel comfortable communicating through videoconferencing. (5) I felt comfortable discussing sensitive topics during videoconferencing sessions. (6) I would be willing to use videoconferencing again. (7) There was no difference between videoconferencing and face-to-face meetings. (8) I was satisfied with the videoconferencing visit as a whole. Questions receiving four or more points were defined as “agree,” and their ratios were calculated and the results are summarized in Table 3.

### Endpoints

The primary endpoint was QOL, which was evaluated using the difference in FACT-G scores at baseline and at the one-month follow-up. The secondary endpoints were depression, prognosis, and patient satisfaction. Depression was evaluated using the difference in CES-D score, and prognosis was evaluated using overall survival (OS). OS was defined as the time from enrollment in the study to death from any cause.

### Statistical analysis

Assuming a two-sided alpha of 0.05, the sample size of 25 enrolled participants in each group (a total of 50 patients) provided at least 80% power to detect at least eight points difference and a common standard deviation of 10 in the FACT-G score. To evaluate patient characteristics, summary statistics were constructed by using frequencies and proportions for categorical data and means and standard deviations for continuous variables. Patient characteristics were compared using the chi-square test or Fisher’s exact test for categorical outcomes and the *t* test for continuous variables. Because this was a nonrandomized clinical trial, with groups assigned according to whether video conferencing can be implemented, background confounding variables need to be adjusted. Analysis of covariance (ANCOVA) was used to adjust for confounding factors between group comparisons of the FACT-G and CES-D. The ANCOVA model included age, sex, PS (0–1, ≥2), primary tumor type (upper and biliopancreatic or lower), presence of a housemate, presence of a house call physician, and presence of the desired online medical treatment. OS was assessed using a log-rank test and summarized using Kaplan–Meier methods. Hazard ratios (HRs) were calculated using Cox proportional hazards regression models. All *p*-values were based on two-tailed hypotheses, and *p* < 0.05 was considered statistically significant. All statistical analyses were performed using the JMP version 15.1.0 software (SAS Institute, NC, USA).

## Results

Overall, 68 patients considered for BSC were screened, of whom 50 were included in this study, and 25 were assigned to each group (excluding 18 patients who could not answer the questionnaire). Data were not available for one patient in each group who failed to attend the second outpatient visit: one patient in the TMD group was hospitalized due to deteriorating general condition, and one patient in the no TMD group died (Fig. 1). In terms of patient background, there were no significant differences between the two groups with respect to age, sex, PS, cancer type, presence of a cohabitant, or home physician’s intervention (Table 1). At baseline, FACT-G scores were 66.44 (43–85) and 66.28 (32–86), *p* = 0.96; the CES-D scores were 15.96 (1–35) and 14.40 (1–30), *p* = 0.48, for the TMD and no TMD groups, respectively, showing no significant differences between the two groups. It was expected that FACT-G and other factors in the baseline would be different for groups with different social backgrounds, but no differences were observed here.

**Table 1.** Baseline Characteristics of Patients in the Study

	Telemedicine group (*n* = 25)	No telemedicine group (*n* = 25)	*p* value
Age, years median (range)	76 (37–86)	78 (47–92)	0.13
Male gender *n* (%)	13 (52)	14 (56)	0.78
ECOG performance status, *n* (%)			
0–1	17 (68)	16 (64)	0.94
≧2	8 (32)	9 (36)	
Type of primary tumor *n* (%)			
Esophageal cancer	3 (12)	3 (12)	0.41
Gastric cancer	2 (8)	3 (12)	
Small bowel cancer	3 (12)	1 (4)	
Colon cancer	10 (40)	8 (32)	
Pancreatic cancer	3 (12)	5 (20)	
Biliary tract cancer	2 (8)	5 (20)	
Others	2 (8)	0	
Housemate *n* (%)	23 (92)	22 (88)	0.63
Visiting doctor *n* (%)	6 (24)	5 (20)	1.00
Initial questionnaire			
FACT-G	66.44 (43–85)	66.28 (32–86)	0.96
CES-D	15.96 (1–35)	14.40 (1–30)	0.48
PPI	2.42 (0–6.5)	2.08 (0–8.0)	0.51

Note: A *t* test for differences in means was used to compare ages and other continuous variables. Categorical variables were compared using chi-square and Fisher’s exact tests.

ECOG, Eastern Cooperative Oncology Group; FACT-G, Functional Assessment of Cancer Therapy-General; CES-D, Center for Epidemiological Studies Depression Scale; PPI, Palliative Prognostic Index.

**FIG. 2.**
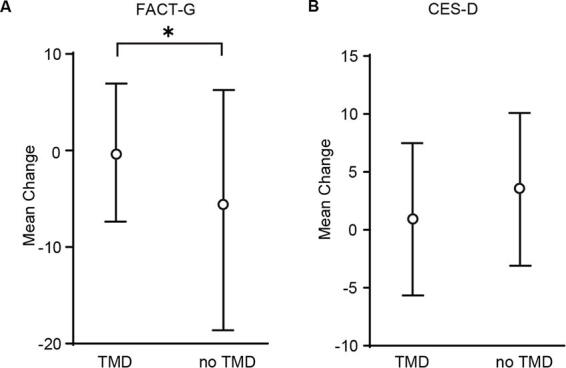
Mean change in QOL and depression from baseline to one month in the two study groups. (**A**) Mean change in FACT-G, (**B**) Mean change in CES-D. QOL, quality-of-life; FACT-G, Functional Assessment of Cancer Therapy-General; CES-D, Center for Epidemiological Studies Depression Scale; TMD, Telemedicine; ^*^p < 0.05, *t* test.

A comparison of measures of QOL at one month showed that the TMD group maintained a better QOL. The difference in FACT-G scores of the one-month follow-up (primary endpoint) from baseline was 0.21 points in the TMD group and 5.96 points in the no TMD group. In the analysis of covariance, the adjusted mean difference was −2.90 (95% CI: −5.76 to 0.034), which was significantly different (*p* = 0.047) (Fig. 2, Table 2).

**Table 2.** Differences in the Initial Questionnaire and That Administered after One Month

	Telemedicine group (mean)	No telemedicine group (mean)	Parameter estimates (95% CI)	*p* value
FACT-G	−0.21	−5.96	−2.90 (−5.76 to 0.034)	*p* = 0.047^*^
CES-D	0.92	3.5	1.09 (−0.86 to 3.04)	*p* = 0.26

^*^ All *p*-values are less than 0.05.

FACT-G, Functional Assessment of Cancer Therapy-General; CI, confidence interval; CES-D, Center for Epidemiological Studies Depression.

**Table 3.** Palliative Videoconference Satisfaction Survey (*n* = 23)

Question	>4 (%)
I could see the other videoconference participants clearly.	100.00
I could hear the other videoconference participants clearly.	86.96
I felt my conversations during the video conferencing session were private.	86.96
I felt comfortable communicating with doctor/nurse through videoconferencing.	95.65
I felt comfortable discussing sensitive topics with doctor/nurse during the videoconference session.	86.96
I would be willing to use videoconferencing again with the doctor/nurse.	91.30
There is no difference between videoconferencing with doctor/nurse and meeting face-to-face.	65.22
I was satisfied with the videoconferencing visit.	91.30

**FIG. 3.**
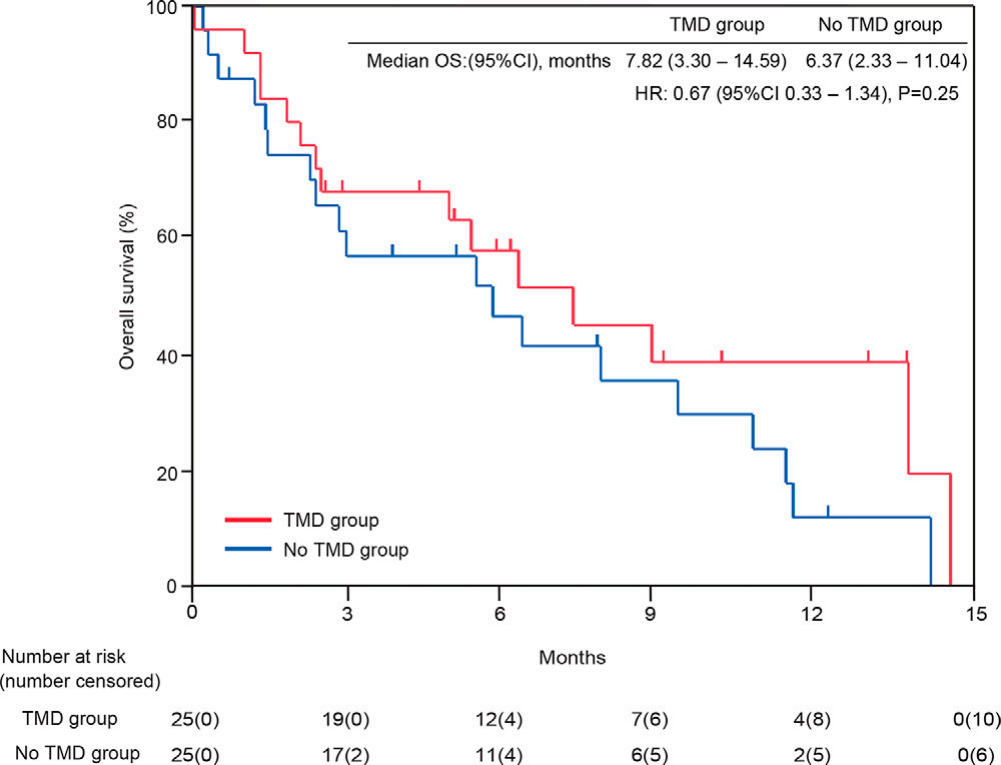
Overall survival in TMD and no TMD groups. TMD, telemedicine; OS, overall survival; HR, hazard ratio.

CES-D (depression indicator) increased by 0.92 points and 3.50 points in the TMD and nonTMD groups, respectively. Lower scores indicated lower depressive tendencies in the TMD group. However, ANCOVA revealed no significant difference between the two groups, with an adjusted mean difference of 1.09 (95% CI: −0.86 to 3.04, *p* = 0.26) (Fig. 2, Table 2). In the TMD and no TMD groups, the median survival time was 7.82 (3.30–14.59) months and 6.37 (2.33–11.04) months, respectively. The HR of 0.67 (95% CI: 0.33–1.34, *p* = 0.25) was not significant between the two groups. (Fig. 3).

The results of the patient satisfaction questionnaire for the TMD group are summarized in Table 3. All patients felt that the facial confirmation was clear. Furthermore, 90% of the patients felt that they could listen to the conversation without any problems and felt comfortable communicating. These findings indicate that video TMD is an effective communication tool. Moreover, 90% of the patients felt that the videoconference was a private space and that they were able to communicate comfortably, including sensitive conversations about death, suggesting that video TMD could be used for palliative care. However, only 65% of patients answered that there was no difference between online and in-person consultations. Previous reports have also shown that while online medical care cannot replace the face-to-face care, it is a reliable, safe and cost-effective method of service delivery while maintaining a high standard of care.^20^ This finding suggests that video TMD may be well accepted when combined with outpatient visits and house calls. Overall, satisfaction with video TMD was high, and many respondents expressed a desire to use online medical care services.

## Discussion

This is one of the few prospective studies to evaluate the usefulness of palliative care combined with TMD via video conferences. The results showed that the TMD group maintained a statistically better QOL with a FACT-G score difference of −0.21 between the baseline and after the intervention compared to −5.96 in the no TMD group, *p* = 0.047. Worsening depression (CES-D) tended to be suppressed in the TMD group; however, the difference was not statistically significant. Additionally, no significant differences were found in OS between the two groups.

Various factors may have contributed to the higher QOL in the TMD group, including the fact that patients felt more secure when in close contact with their attending doctors through online medical services. A previous telehealth study in Japan found that easy access to physicians led to a sense of security among patients.^21^ Particularly, patients with cancer often have difficulty visiting hospitals due to deterioration in their general condition, and it is thought that easing physical barriers through online medical care will have a positive effect on patients.^22^ Additionally, the attending physician could examine and perform ACP for patients while seeing them at home. When patients visit a hospital, they may forget some of their complaints and/or may not be able to talk to their doctors about these issues.^23^ Moreover, there are many situations in which patients are unable to express themselves clearly because of tension in outpatient consultation booths. In a familiar environment surrounded by family, patients can communicate in a relaxed state.^24,25^ It was expected that some patients would feel more comfortable talking about sensitive topics such as end-of-life policies in face-to-face conversations, while others would feel more comfortable talking about them remotely through video screens.^26^ However, as shown in the results of the patient satisfaction evaluation, most patients (approximately 90%) were able to discuss such topics without any hindrance. Other advantages of online palliative care are that counseling and symptom monitoring can be provided in a cost-effective manner, which may lead to improved adherence, increased referral to hospice care, and fewer visits to acute care unit.^27,28^ In this study, there was one case in which deterioration of the patient’s condition was detected through online medical care, and the patient was admitted to a hospital.

CES-D tended to be increased greater in the no TMD group (3.50 points) than the TMD group (0.92 points), and TMD might prevent worsening of depression. However, there was no significant difference. Depression is less likely to benefit from online medical care than QOL. The lack of significant differences may be due to the low number of times of TMD, short follow-up period and insufficient sample size. Previous studies examining the effect of online medical care on depression have used multiple visits, and differences may be seen when long-term follow-up was conducted.^29,30^ In terms of OS, the TMD group appears to be longer; however, there is no significant difference. This may have been due to the fact that the online palliative care provider was able to detect and deal with deteriorating conditions before they worsened. The patient was able to check for medication overdose according to the situation at home, and one patient was actually able to make a decision to be hospitalized before the situation worsened. The sample size was sufficient for the power of the primary outcome, QOL, but not for OS, and studies in settings with larger populations are needed.

Some patients pointed out problems related to hardware, such as equipment and Internet environment. In many cases, the use of smartphones and other devices is limited in elderly patients. In previous studies, there were cases in which technical frustrations, such as the inability to use devices^31,32^ and lack of Internet access, such as Wi-Fi, were problematic in patients.^33,34^ Although those who had support from their children were able to use these devices, accessing TMD may be difficult for elderly couples with no family or caregiver nearby. The number of elderly people familiar with the use of ICT devices is gradually increasing, and the devices are expected to gradually penetrate the elderly population over time.^35^

This study is an examination of TMD in combination with usual care, not TMD alone. In the area of palliative care, TMD-only studies may have ethical issues, and an add-on setting such as this one may be useful. In addition, the timing of online medical care for cancer patients need to be discussed. In recent years, it has been recommended that palliative care be introduced at an earlier timing,^2^ but for patients undergoing chemotherapy, visits to the hospital for intravenous infusion administration are mandatory and may be better in the form of mainly face-to-face consultations because of the high frequency of visits. On the contrary, patients who have terminated chemotherapy, such as those in this study, no longer need to go to the hospital for intravenous infusions. For such patients, TMD may provide more effective palliative care.

This study has some limitations. First, the study was not randomized. Some patients did not have a videoconferencing device or family support to use the device, making it difficult to assign patients to the TMD group by pure randomization. Nonrandomization excluded the possibility to evaluate the influence of social contacts because patients with high social contact may gather in the TMD group. It was conceivable that this may have contributed to the maintenance of QOL. It was also thought that patients might be younger or have more support from their family members, etc., but no differences in background, including age, PS, disease, presence of family members living with the patient, and presence of home physician intervention were found this time. Additionally, analyses of FACT-G and CES-D differences were performed using ANCOVA to address bias as mentioned in the material and methods part. Second, this study was conducted at a single institution, which may have introduced bias through the study population. We believe that multicenter prospective studies with larger sample sizes are required to evaluate the findings of this study more accurately. Third, the underlying circumstance of this study period, the COVID-19 epidemic, may have influenced the results. Online medical care that is not face-to-face may have had a positive impact on patient satisfaction and other evaluations during the COVID-19 epidemic. Lastly, socio-economic status (SES) may have differed in the two groups, and SES may have affected QoL outcomes. Otherwise, if SES had a greater impact, the preintervention values would also change, but the baseline FACT-G values in this study showed no differences between groups.

## Conclusion

We found that the combination of video TMD and outpatient treatment is effective in maintaining QOL in advanced cancer patients and is feasible based on patient satisfaction. It is desirable to further evaluate the usefulness of palliative care using TMD and establish its clinical significance.

## Notes

**Cite this article as:** Chida A, Hamamoto Y, Hirata K, Sato Y, So E, Kishimoto S, Noguchi S, Horie S, Saito Y, Shimozaki K, Tsugaru K, Togasaki K, Kawasaki K, Hayashi H, Kanai T (2024) The effectiveness and feasibility of palliative care via video telemedicine for patients with advanced cancer: a nonrandomized prospective clinical trial comparing combination of telemedicine and in-person care with in-person care alone, *Palliative Medicine Reports* 5:1, 503–511, DOI: 10.1089/pmr.2024.0053.

## Abbreviations Used

CES-D: Center for Epidemiological Studies Depression Scale
ECOG PS: Eastern Cooperative Oncology Group Performance Status
FACT-G: Functional Assessment of Cancer Therapy-General
ICT: Information and Communication Technology
OS: Overall Survival
PPI: Palliative Prognostic Index
QOL: Quality of Life
TMD: Telemedicine

## References

[1] Temel JS, Greer JA, El-Jawahri A, et al. Effects of early integrated palliative care in patients with Lung and GI Cancer: A randomized clinical trial. J Clin Oncol 2017;35(8):834–841; doi: 10.1200/jco.2016.70.5046 28029308 PMC5455686

[2] Temel JS, Greer JA, Muzikansky A, et al. Early palliative care for patients with metastatic non-small-cell lung cancer. N Engl J Med 2010;363(8):733–742; doi: 10.1056/NEJMoa1000678 20818875

[3] Prescott AT, Hull JG, Dionne-Odom JN, et al. The role of a palliative care intervention in moderating the relationship between depression and survival among individuals with advanced cancer. Health Psychol 2017;36(12):1140–1146; doi: 10.1037/hea0000544 29048177 PMC5709150

[4] Morita T, Tsunoda J, Inoue S, et al. The palliative prognostic index: A scoring system for survival prediction of terminally ill cancer patients. Support Care Cancer 1999;7(3):128–133; doi: 10.1007/s005200050242 10335930

[5] Sánchez-Cárdenas MA, Iriarte-Aristizábal MF, León-Delgado MX, et al. Rural palliative care telemedicine for advanced cancer patients: A systematic review. Am J Hosp Palliat Care 2023;40(8):936–944; doi: 10.1177/10499091221130329 36331174

[6] Hancock S, Preston N, Jones H, et al. Telehealth in palliative care is being described but not evaluated: A systematic review. BMC Palliat Care 2019;18(1):114; 5. 31835998 10.1186/s12904-019-0495-5 PMC6911274

[7] Sinclair C, Holloway K, Riley G, et al. Online mental health resources in rural Australia: Clinician perceptions of acceptability. J Med Internet Res 2013;15(9):e193; doi: 10.2196/jmir.2772 24007949 PMC3785998

[8] Jiang S, Xiang J, Gao X, et al. The comparison of telerehabilitation and face-to-face rehabilitation after total knee arthroplasty: A systematic review and meta-analysis. J Telemed Telecare 2018;24(4):257–262; doi: 10.1177/1357633x16686748 28027679

[9] Basch E, Deal AM, Kris MG, et al. Symptom monitoring with patient-reported outcomes during routine cancer treatment: A randomized controlled trial. J Clin Oncol 2016;34(6):557–565; doi: 10.1200/jco.2015.63.0830 26644527 PMC4872028

[10] Tang M and Reddy A. Telemedicine and its past, present, and future roles in providing palliative care to advanced cancer patients. Cancers (Basel) 2022;14(8); doi: 10.3390/cancers14081884 PMC9032063 35454791

[11] Chua IS, Olmsted M, Plotke R, et al. Video and In-Person Palliative Care Delivery Challenges before and during the COVID-19 Pandemic. J Pain Symptom Manage 2022;64(6):577–587; doi: 10.1016/j.jpainsymman.2022.08.005 35985551 PMC9383956

[12] Kaya E, Lewin W, Frost D, et al. Scalable model for delivery of inpatient palliative care during a pandemic. Am J Hosp Palliat Care 2021;38(7):877–882; doi: 10.1177/10499091211005701 33823653 PMC8135235

[13] Narvaez RA, Canaria A, Nifras SR, et al. The role of telehealth on quality of life of palliative care patients during the COVID-19 pandemic: An integrative review. Int J Palliat Nurs 2022;28(12):583–589; doi: 10.12968/ijpn.2022.28.12.583 36520100

[14] Jess M, Timm H, and Dieperink KB. Video consultations in palliative care: A systematic integrative review. Palliat Med 2019;33(8):942–958; doi: 10.1177/0269216319854938 31187688

[15] Mathews JJ, Chow R, Wennberg E, et al. Telehealth palliative care interventions for patients with advanced cancer: A scoping review. Support Care Cancer 2023;31(8):451; doi: 10.1007/s00520-023-07907-z 37421447

[16] Cella DF, Tulsky DS, Gray G, et al. The functional assessment of cancer therapy scale: Development and validation of the general measure. J Clin Oncol 1993;11(3):570–579; doi: 10.1200/jco.1993.11.3.570 8445433

[17] Fumimoto H, Kobayashi K, Chang CH, et al. Cross-cultural validation of an international questionnaire, the General Measure of the Functional Assessment of Cancer Therapy scale (FACT-G), for Japanese. Qual Life Res 2001;10(8):701–709; doi: 10.1023/a:1013851216181 11871591

[18] Radloff LS. The CES-D Scale: A self-report depression scale for research in the general population. Psychological Measurement 1977;1(3):385–401.

[19] Perri GA, Abdel-Malek N, Bandali A, et al. Early integration of palliative care in a long-term care home: A telemedicine feasibility pilot study. Palliat Support Care 2020;18(4):460–467; doi: 10.1017/s1478951520000012 32066517

[20] Sutherland AE, Stickland J, and Wee B. Can video consultations replace face-to-face interviews? Palliative medicine and the Covid-19 pandemic: Rapid review. BMJ Support Palliat Care 2020;10(3):271–275; doi: 10.1136/bmjspcare-2020-002326 PMC7295858 32457086

[21] Aoki N, Ohta S, Yamamoto H, et al. Triangulation analysis of tele-palliative care implementation in a rural community area in Japan. Telemed J E Health 2006;12(6):655–662; doi: 10.1089/tmj.2006.12.655 17250487

[22] Reddy A, Arthur J, Dalal S, et al. Rapid transition to virtual care during the COVID-19 Epidemic: Experience of a supportive care clinic at a tertiary care cancer center. J Palliat Med 2021;24(10):1467–1473; doi: 10.1089/jpm.2020.0737 33535019

[23] Kwame A and Petrucka PM. A literature-based study of patient-centered care and communication in nurse-patient interactions: Barriers, facilitators, and the way forward. BMC Nurs 2021;20(1):158; doi: 10.1186/s12912-021-00684-2 34479560 PMC8414690

[24] Sandsdalen T, Hov R, Høye S, et al. Patients’ preferences in palliative care: A systematic mixed studies review. Palliat Med 2015;29(5):399–419; doi: 10.1177/0269216314557882 25680380

[25] Feliciano DR and Reis-Pina P. Enhancing End-of-Life Care with home-based palliative interventions: A systematic review. J Pain Symptom Manage 2024; doi: 10.1016/j.jpainsymman.2024.07.005 39002710

[26] Nguyen M, Waller M, Pandya A, et al. A review of patient and provider satisfaction with telemedicine. Curr Allergy Asthma Rep 2020;20(11):72; doi: 10.1007/s11882-020-00969-7 32959158 PMC7505720

[27] Riggs A, Breuer B, Dhingra L, et al. Hospice enrollment after referral to community-based, specialist palliative care: Impact of telephonic outreach. J Pain Symptom Manage 2017;54(2):219–225; doi: 10.1016/j.jpainsymman.2017.03.007 28450217

[28] Dilhani WNS, Mitchell S, Dale J, et al. A mixed-methods systematic review investigating the use of digital health interventions to provide palliative and end-of-life care for patients in low- and middle-income countries. Palliat Care Soc Pract 2024;18:26323524241236965; doi: 10.1177/26323524241236965 38617095 PMC11010586

[29] Bolier L, Haverman M, Kramer J, et al. An Internet-based intervention to promote mental fitness for mildly depressed adults: Randomized controlled trial. J Med Internet Res 2013;15(9):e200; doi: 10.2196/jmir.2603 24041479 PMC3929047

[30] Egede LE, Acierno R, Knapp RG, et al. Psychotherapy for depression in older veterans via telemedicine: A randomised, open-label, non-inferiority trial. Lancet Psychiatry 2015;2(8):693–701; doi: 10.1016/s2215-0366(15)00122-4 26249300

[31] Brody AA, Convery KA, Kline DM, et al. Transitioning to remote recruitment and intervention: A tale of two palliative care research Studies Enrolling Underserved Populations During COVID-19. J Pain Symptom Manage 2022;63(1):151–159; doi: 10.1016/j.jpainsymman.2021.06.017 34161811 PMC8685301

[32] Leite HH, Hodgkinson IR, and Gruber T. New development: ‘Healing at a distance’—telemedicine and COVID-19. Public Money Manag 2020;40(6):483–485.

[33] Lopez AM, Lam K, and Thota R. Barriers and facilitators to telemedicine: Can you hear me now? Am Soc Clin Oncol Educ Book 2021;41:25–36; doi: 10.1200/edbk_320827 34010056

[34] Wosik J, Fudim M, Cameron B, et al. Telehealth transformation: COVID-19 and the rise of virtual care. J Am Med Inform Assoc 2020;27(6):957–962; doi: 10.1093/jamia/ocaa067 32311034 PMC7188147

[35] Scott Kruse C, Karem P, Shifflett K, et al. Evaluating barriers to adopting telemedicine worldwide: A systematic review. J Telemed Telecare 2018;24(1):4–12; doi: 10.1177/1357633x16674087 29320966 PMC5768250

